# Effects of hyperoxia on dynamic muscular endurance are associated with individual whole-body endurance capacity

**DOI:** 10.1371/journal.pone.0231643

**Published:** 2020-04-21

**Authors:** Yuta Kojima, Chiho Fukusaki, Naokata Ishii

**Affiliations:** 1 Department of Human and Engineered Environmental Studies, Graduate School of Frontier Sciences, The University of Tokyo, Kashiwa, Chiba, Japan; 2 Research Center for Total Life Health and Sports Sciences, Graduate School of Frontier Sciences, The University of Tokyo, Kashiwa, Chiba, Japan; 3 Department of Life Sciences, Graduate School of Arts and Sciences, The University of Tokyo, Meguro, Tokyo, Japan; University of Calgary, CANADA

## Abstract

Low-intensity training involving high repetitions is recommended to enhance muscular endurance. Hyperoxic conditions could increase the number of repetitions until exhaustion and thereby improve the results of muscular endurance training. This study aimed to investigate the acute effects of hyperoxia on dynamic muscular endurance, and determine individual factors that may be related to these effects. A single-blinded, counterbalanced crossover design was used. Twenty-five young men performed repetitions of the one-arm preacher curl at 30% of their 1-repetition maximum until exhaustion under hyperoxic and normoxic conditions. The maximum number of repetitions was recorded as an index of muscular endurance. Electromyogram (EMG) and near-infrared spectroscopy parameters were measured in the biceps brachii. The maximum number of repetitions was greater (*P* < 0.001) under hyperoxic conditions (132 ± 59 repetitions) than under normoxic conditions (114 ± 40 repetitions). The root mean square amplitude of EMG and oxygenated hemoglobin concentration for the last five repetitions under normoxic conditions were greater than those under hyperoxic conditions (*P* = 0.015 and *P* = 0.003, respectively). The percent change in the maximum number of repetitions between hyperoxic and normoxic conditions had significant positive correlations with individual maximal oxygen uptake measured using an incremental cycle ergometer test (*r* = 0.562, 95% confidence intervals [CI] = 0.213–0.783, *P* = 0.003), but not with muscle strength (*τ* = −0.124, 95% CI = −0.424–0.170, *P* = 0.387). The 95% CI for the correlation coefficient between the percent change in the maximum number of repetitions and muscular endurance included 0 (*τ* = 0.284, 95% CI = −0.003–0.565, *P* = 0.047); this indicated no significant correlation between the two parameters. The results suggest that hyperoxia can acutely enhance dynamic muscular endurance, with delayed elevation of EMG amplitude due to fatigue, and the effects are associated with individual whole-body endurance capacity.

## Introduction

Muscular endurance is defined as the ability of a muscle group to execute repeated contractions over a period of time sufficient to cause muscular fatigue, or to maintain a specific percentage of maximum voluntary contraction for a prolonged period of time [1]. Low-intensity training involving high repetitions is generally recommended to enhance muscular endurance [1]. Most previous studies explaining the rationale for low-intensity training involving high repetitions for muscular endurance used repetitions to the point of exhaustion [2,3]; therefore, increasing the number of repetitions performed to the point of exhaustion might be effective for improving muscular endurance. As a way to increase the number of repetitions until exhaustion, we focused on exercise performed under hyperoxic conditions.

Several studies have investigated the effects of hyperoxia on exercise performance. Measures of maximal aerobic exercise performance, such as maximal oxygen uptake (${\dot{V}O}_{2max}$) [4,5] and maximum exercise duration in high-intensity aerobic exercise [6,7], increase when exercise is performed under hyperoxic conditions. Hyperoxic conditions also increase maximal anaerobic power [8]. During sub-maximal aerobic exercise, the heart rate (HR) [9], blood lactate concentration [9,10], and rating of perceived exertion (RPE) [11] are lower under hyperoxic conditions than normoxic conditions. Thus, many previous studies have reported positive effects of hyperoxia during whole-body aerobic and anaerobic exercise. These advantages of hyperoxic conditions are provided mainly by higher oxygen delivery to muscles [4,12,13] and changes in muscle metabolism [10,14] compared to normoxic conditions. Meanwhile, only a few studies have evaluated the effects of hyperoxia on local muscular endurance, and these studies reported that the mean value of peak torque during 60 maximal knee extensions was higher under hyperoxic conditions than normoxic conditions [15,16], thus suggesting positive effects of hyperoxia on muscular endurance. Although these previous studies used maximum voluntary contractions as the muscular endurance exercise, low-intensity repetitive exercise is recommended for appropriate muscular endurance training [1]. To the best of our knowledge, no reports have investigated the effects of hyperoxia on low-intensity repetitive muscle contractions.

One of the determining factors for muscular endurance is oxygen supply to the exercising muscles [17]. Many studies have reported that the arterial blood oxygen content (CaO_2_) and/or arterial blood oxygen saturation exhibit greater increases under hyperoxic conditions than under normoxic conditions [4,12,13,18], indicating a potential advantage of hyperoxic conditions in terms of increased oxygen supply to the exercising muscles during muscular endurance exercise. Previous studies showing positive effects of hyperoxia on exercise suggested some possible underlying mechanisms that included attenuated degradation of phosphocreatine [14], reduced muscle glycogenolysis [10], and faster phosphocreatine resynthesis [15]. These possible underlying mechanisms, although revealed during exercises other than low-intensity repetitive exercise, are associated with energy metabolism in muscles and might positively affect low-intensity repetitive muscle contractions.

Previous studies have indicated that the effects of hyperoxia on exercise depend on the physical characteristics of the individuals [19–21]; those with arterial oxygen desaturation during maximal aerobic exercise (exercise-induced arterial hypoxemia) demonstrated larger increases in ${\dot{V}O}_{2max}$ under hyperoxic conditions than did those with no or less arterial oxygen desaturation [19,20]. Moreover, it was reported that the effects of oxygen supply during aerobic exercise were not the same for all patients with obstructive pulmonary disease; some patients responded positively, some responded negatively, and some were non-responders [21]. To the best of our knowledge, no study has investigated individual differences in the effects of hyperoxia on muscular endurance.

The primary purpose of this study was to investigate the effects of hyperoxia on muscular endurance during low-intensity single-joint repetitive muscle contractions until exhaustion. The hypothesis was that muscular endurance would improve under hyperoxic conditions, even if the exercise involved low-intensity single-joint repetitive muscle contractions. We also aimed to identify individual differences in the effects of hyperoxia on muscular endurance. Exercise-induced arterial hypoxemia tends to occur in individuals with a high ${\dot{V}O}_{2max}$ [22] and can be associated with the effects of hyperoxia in healthy subjects [19,20]. Furthermore, oxygen supply to the exercising muscle is a determining factor for muscular endurance [17] and individuals with a high ${\dot{V}O}_{2max}$ have a high ability to transport oxygen to the exercising muscle [23]. Therefore, we hypothesized that there would be individual differences in the effects of hyperoxia on muscular endurance, and that the differences would depend on the individual’s physical fitness, particularly the ${\dot{V}O}_{2max}$.

## Materials and methods

### Participants

Twenty-five healthy and active young men with no formal physical training participated in this study. None had a history of cardiorespiratory diseases or musculoskeletal disorders. The age, height, and mass of the subjects were 24 ± 2 years, 173.2 ± 5.8 cm, and 66.1 ± 7.5 kg (means ± standard deviations [SD]), respectively. This study was designed and conducted in accordance with the Declaration of Helsinki and approved by the Research Ethics Committee of the University of Tokyo (Approval No. 14–66). Prior to the commencement of this study, written informed consent was obtained from all participants.

### Study designs and procedures

**Fig 1:**
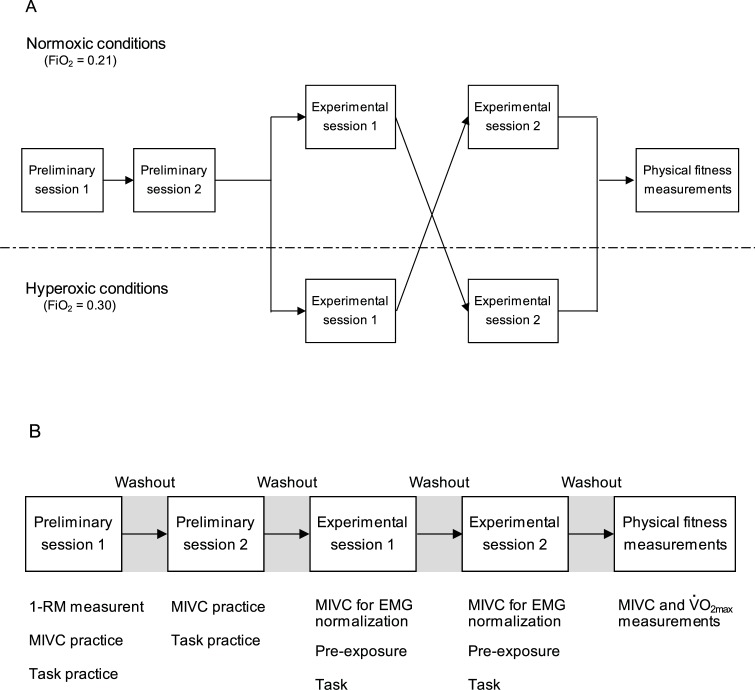
Experimental design.

This study used a single-blinded, counterbalanced crossover design to identify the acute effects of hyperoxia on muscular endurance (Fig 1A). Individual differences in the effects of hyperoxia among participants were evaluated as the percent change in muscular endurance between hyperoxic conditions and normoxic conditions. Correlations between the percent change in muscular endurance and physical fitness parameters were calculated to identify factors related to the acute effects of hyperoxia on muscular endurance. Cardiorespiratory fitness (${\dot{V}O}_{2max}$), muscular endurance, and muscle strength were defined as physical fitness parameters.

This study consisted of two preliminary sessions, two experimental sessions, and physical fitness measurements (Fig 1B). In the first preliminary session, one-repetition maximum (1-RM) measurement, and practice of maximum isometric voluntary contraction (MIVC) and a single-joint repeated weight-lifting task were performed using the preferred arm (right arm for all subjects). The preferred arm was determined using the short form of the Edinburgh Handedness Inventory [24]. In the second preliminary session, the subjects practiced the MIVC and the single-joint repeated weight-lifting task again for further familiarization. These two preliminary sessions were conducted under normobaric, normoxic conditions (NOX; fractional inspired oxygen concentration [FiO_2_] = 0.21) with a rest period of at least 4 days to avoid muscle soreness or fatigue.

A, Flow chart of the study protocol and experimental conditions. The boxes above and below the broken line indicate that these sessions were conducted under normoxic and hyperoxic conditions, respectively. B, Details regarding the implementation of each session. The task was one-arm preacher curl at an intensity of 30% of the one-repetition maximum to the point of exhaustion. FiO_2_, fractional inspired oxygen concentration; 1-RM, one-repetition maximum; MIVC, maximum isometric voluntary contraction; EMG, electromyogram; ${\dot{V}O}_{2max}$, maximal oxygen uptake.

Six to 8 days after the second preliminary session, the first experimental session was conducted. The experimental session was performed twice under two oxygen conditions in an environmental simulation chamber (232 cm W × 240 cm H × 228 cm D): once under normobaric, hyperoxic conditions (HOX; FiO_2_ = 0.30), and once under NOX. The HOX were created using an oxygen generator (TOK-12DX; IBS, Osaka, Japan) that produced high-purity oxygen from air through synthetic zeolite-based sorbents via pressure swing adsorption. During the experimental session under NOX, a compressor (Emeraude ES-4AD-5; Kobe Steel, Hyogo, Japan) injected air into the chamber. We determined FiO_2_ = 0.30 as the oxygen concentration in HOX. Previous studies evaluating the effects of hyperoxia on muscular endurance using maximal knee contractions [15,16] were conducted under higher oxygen conditions (FiO_2_ = 1.00 at 1.3 ATA and FiO_2_ = 0.99 at 1.0 ATA); no study has used hyperoxic conditions as mild as this study. A systematic review [25] suggested that FiO_2_ ≥ 0.30 is enough to increase general exercise performance, including time trials and time to exhaustion of aerobic exercise, and dynamic contractions of large locomotory muscle. Considering the feasibility of hyperoxic conditions in a practical exercise environment in terms of generating hyperoxic conditions per se and safety against fire and oxygen toxicity, we chose FiO_2_ = 0.30 in this study. The two experimental sessions were conducted 6 days apart. The experimental sessions consisted of the MIVC measurement, a 30-min exposure (pre-exposure), a 1-min warm-up, and one set of a single-joint repeated weight-lifting task until exhaustion. The pre-exposure, warm-up, and task were conducted in the chamber. The subjects were instructed to sleep at least 6 h the night before the experiment, to refrain from alcohol consumption and hard exercise for 24 h, and to refrain from food or caffeine ingestion for 3 h before starting each session. We confirmed that each participant adhered to these restrictions before starting each experimental session. Physical fitness measurements were performed approximately 1 week after the second experimental session.

The 1-RM measurement of the one-arm preacher curl followed the protocol of a previous study [26] to determine the exercise load for the experimental sessions. The one-arm preacher curl was performed using a dumbbell while sitting on a preacher curl bench (GFB350; Body-Solid Inc., Illinois, USA). The range of motion was set from 0 to 120° of elbow flexion. An elbow joint angle of 0° was determined as the position of the forearm on the preacher curl bench. Before performing the 1-RM measurement, each subject bent their elbow at a 120° angle while the elbow angle was measured with a goniometer (CK-S4305-300; Chin Kou Medical Instrument, New Taipei, Taiwan); a thin bar was positioned to touch the subject’s wrist to mark the limit of elbow flexion (120° angle). The MIVC measurement was conducted using a dynamometer (Myoret RZ-450; Kawasaki Heavy Industries, Hyogo, Japan). The subjects were encouraged to exert maximal isometric strength of the elbow flexor muscles at 90° of elbow flexion. An electromyogram (EMG) signal at peak MIVC was obtained to normalize the EMG during the single-joint repeated weight-lifting task. In the warm-up, subjects repeated the preacher curl movement (elbow flexion in 1 s and extension in 1 s in accordance with the rhythm of a metronome) without load for 1 min. In the single-joint repeated weight-lifting task, subjects performed one set of one-arm preacher curls at an intensity of 30% of 1-RM until exhaustion. The one-arm preacher curl was conducted in a manner similar to that of the 1-RM measurement (using a dumbbell; range of motion, 0–120°) and the warm-up (elbow flexion in 1 s and extension in 1 s in accordance with a metronome). The point when subjects could no longer perform elbow flexion and extension in the fixed range and rhythm was defined as the point of exhaustion and the maximum number of repetitions until exhaustion was measured using a counter.

### Physiological measurements

Surface EMG, tissue oxygenation level, HR, and blood lactate concentration were measured during the single-joint repeated weight-lifting task. The surface EMG activities of the right biceps brachii muscle were recorded using bipolar surface electrodes (SX230; Biometrics, Newport, UK) during the MIVC and single-joint repeated weight-lifting task. After skin preparation, the electrodes were placed on the short head of the biceps brachii along the muscle fibers with an inter-electrode distance of 20 mm. The EMG signal was amplified 1000 times (SX230; Biometrics), digitized at a frequency of 2000 Hz (PowerLab 16/35; ADInstruments, Castle Hill, Australia), and recorded on a personal computer.

To estimate the tissue oxygenation level, the changes in the concentration (μmol·L^-1^) of oxygenated hemoglobin (ΔO_2_Hb), deoxygenated hemoglobin (ΔHHb), and total hemoglobin (ΔcHb) from baseline were measured continuously during the single-joint repeated weight-lifting task using near-infrared spectroscopy (NIRS; NIRO 200; Hamamatsu Photonics, Shizuoka, Japan). After skin preparation, the optical probe was positioned over the long head of the right biceps brachii with the distance of 40 mm between the light emitter and the detector. The probe was firmly attached using double-sided tape and covered with black rubber tape. The baseline value was obtained at the start of the pre-exposure. The NIRS data were collected at 1 Hz.

HR was recorded continuously at an interval of 5 s using a HR monitor (s810i; Polar Electro Oy, Kempele, Finland), starting 1 min before the pre-exposure and continuing until the end of the single-joint repeated weight-lifting task. The sensor was attached to the chest.

To measure the blood lactate concentration, approximately 5 μL of blood was obtained from the left fingertip and analyzed using a portable blood lactate analyzer (Lactate Pro LT-1710; ARKRAY, Kyoto, Japan). The blood samples were collected after the pre-exposure (before starting the task) and 2 min after the single-joint repeated weight-lifting task.

### Measurements of physical fitness

The physical fitness measurements consisted of specific muscle strength and ${\dot{V}O}_{2max}$ measurements. These measurements were conducted under normal atmospheric (normobaric, normoxic) conditions. The maximum number of repetitions in the single-joint repeated weight-lifting task under NOX was used as an index of muscular endurance. To estimate muscle strength, maximal isometric elbow flexion strength was measured in a manner similar to that of the MIVC measurement in the experimental sessions. For this muscle strength measurement, no EMG electrodes were attached to the subject unlike the MIVC measurement. After the muscle strength measurement, an incremental exercise test was conducted using a cycle ergometer (Aerobike 75XL III; Konami Sports Club, Tokyo, Japan) to measure ${\dot{V}O}_{2max}$ as an index of cardiorespiratory fitness. After 3 min of ergocycle exercise at 25 W, the workload was increased in a ramp fashion (25 W/min) until exhaustion. During exercise, oxygen uptake (${\dot{V}O}_{2}$) and carbon dioxide production (${\dot{V}CO}_{2}$) were measured utilizing a gas analyzer (Aeromonitor AE-300S; Minato Medical Science, Osaka, Japan) using a breath-by-breath method. HR was measured using standard bipolar-lead electrocardiography (Life Scope I; Nihon Kohden, Tokyo, Japan). RPE was measured at the end of the exercise using a Borg scale [27]. ${\dot{V}O}_{2max}$ was calculated as the mean of 30 s of data that met at least three of the following four criteria [28,29]: (1) ${\dot{V}O}_{2}$ reached a plateau with increasing work rate, (2) respiratory exchange ratio (${\dot{V}CO}_{2}$/${\dot{V}O}_{2}$) ≥ 1.1, (3) HR ± 10 bpm of age-predicted maximal HR calculated by the formula (220 –age), and (4) RPE ≥ 19.

### Data analyses

The percent change in the maximum number of repetitions between HOX and NOX was calculated using the following formula: (HOX_max_ − NOX_max_) / NOX_max_ × 100, where HOX_max_ and NOX_max_ indicate the maximum number of repetitions under HOX and NOX, respectively.

To estimate the muscle activation level, the root mean square (RMS) amplitude of the EMG was calculated and expressed relative to the peak EMG RMS amplitude during MIVC measurement (% EMG_MIVC_). The peak EMG RMS amplitude was measured as the RMS for 0.5 s (0.25 s on each side of peak torque). The RMS amplitude of the EMG during the single-joint repeated weight-lifting task was calculated over five repetitions (10-s time window) and the RMS values for the first, middle, and last five repetitions under NOX (0% NOX_max_, 50% NOX_max_, and 100% NOX_max_, respectively) were compared to those for the same repetition numbers under HOX. For example, if a subject’s maximum number of repetitions under NOX was 101, then the first, middle, and last five repetition numbers were 1–5, 49–53, and 97–101, respectively, and the values for these repetition numbers under NOX were compared to those under HOX. The last five repetitions under HOX (100% HOX_max_) were also averaged and compared to the HOX value at 100% NOX_max_. These analyses were applied to the 22 subjects because the maximum number of repetitions under HOX was smaller than that under NOX in three subjects. The NIRS and HR data were analyzed in the same manner as that used for the EMG data.

### Statistical analyses

Normality of the data was analyzed using the Shapiro-Wilk test. A nonparametric Wilcoxon signed-rank test was used to test for differences in the maximum number of repetitions between HOX and NOX (n = 25). A two-way repeated-measures analysis of variance (ANOVA) was performed to estimate the oxygen concentration effect (HOX and NOX), time effect (each measurement point), and interaction effect in the RMS amplitude of EMG, ΔO_2_Hb, ΔHHb, ΔcHb, HR, and blood lactate concentration (n = 22). ΔHHb, HR, and blood lactate concentration were judged as non-normally distributed and logarithmically transformed prior to the ANOVA test. When significant oxygen concentration and/or interaction effects were found, a post hoc test was conducted using a paired *t*-test with a Bonferroni correction between HOX and NOX values at each measurement point (n = 22). Additionally, a paired *t*-test was used to test for differences between the HOX values at 100% HOX_max_ and 100% NOX_max_ in the RMS amplitude of EMG, ΔO_2_Hb, ΔHHb, ΔcHb, and HR (n = 22). A paired *t*-test was conducted with a two-tailed test of significance. Correlations between the percent change in the maximum number of repetitions and the physical fitness indices (${\dot{V}O}_{2max}$, maximum number of repetitions, and maximal isometric elbow flexion strength) were calculated using Pearson’s correlation coefficient (${\dot{V}O}_{2max}$; n = 25) and Kendall’s rank correlation coefficient (maximum number of repetitions and maximal isometric elbow flexion strength; n = 25).

Effect size was analyzed by calculating *r* for a Wilcoxon signed-rank test, Cohen’s *d* for a paired *t*-test, and partial *η*^2^ for a two-way ANOVA. Data are expressed as the mean ± SD, and *P* < 0.05 was considered statistically significant. For a paired *t*-test with a Bonferroni correction, *P* < 0.017 was considered significant. The statistical analyses were performed using IBM SPSS Statistics 21.0 (SPSS Japan, an IBM Company, Tokyo, Japan) except for the calculation of effect size *r* and Cohen’s *d* values using G*Power 3 Ver.3.1.9.2 [30] and 95% confidence intervals (CI) using R software (R-3.6.2; R Foundation for Statistical Computing, Vienna, Austria).

## Results

The maximum number of repetitions under HOX (132 ± 59 repetitions) was significantly larger than under NOX (114 ± 40 repetitions; *P* < 0.001, *r* = 0.777).

**Fig 2:**
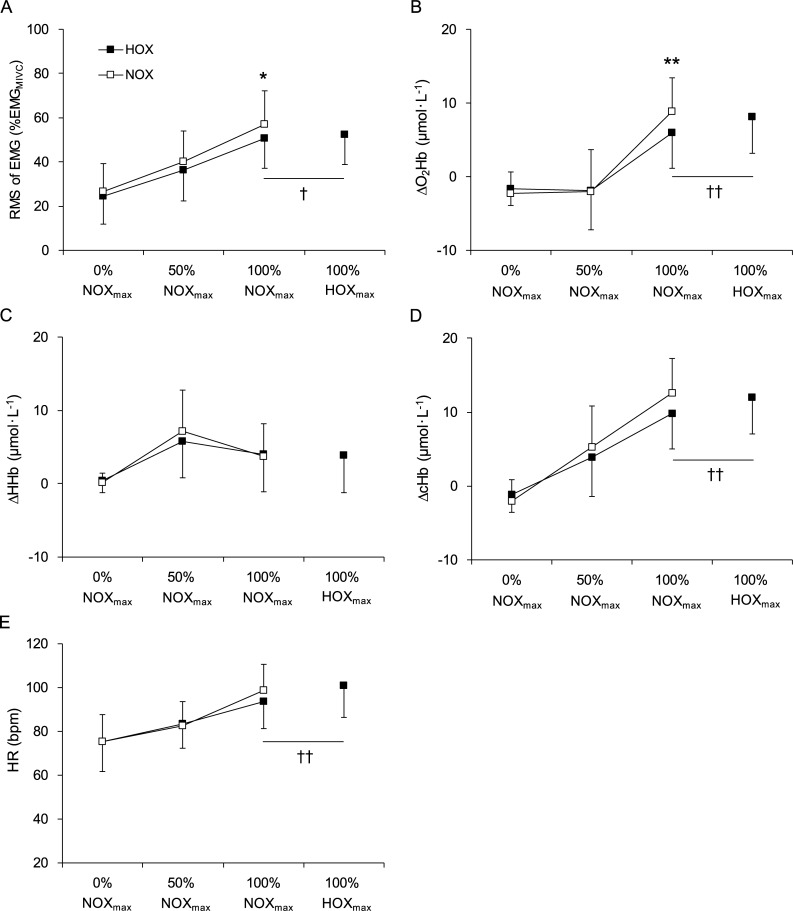
Physiological indices during a single-joint repeated weight-lifting task under hyperoxic and normoxic conditions (n = 22). A, Root mean square amplitude of electromyogram (RMS of EMG) during a single-joint repeated weight-lifting task under hyperoxic (HOX) and normoxic (NOX) conditions. B, Change in oxygenated hemoglobin concentration (ΔO_2_Hb). C, Change in deoxygenated hemoglobin concentration (ΔHHb). D, Change in total hemoglobin concentration (ΔcHb). E, Heart rate (HR). The x-axis indicates the time course during the task. NOX_max_ represents the maximum number of repetitions under NOX. 0% NOX_max_, 50% NOX_max_, and 100% NOX_max_ represent the first, middle, and last five repetitions under NOX, respectively. HOX_max_ represents the maximum number of repetitions under HOX. 100% HOX_max_ represents the last five repetitions under HOX. Error bars indicate standard deviation. *Significant difference (*P* < 0.017); **Significant difference (*P* < 0.003) between NOX and HOX. †Significant difference (*P* < 0.05); ††Significant difference (*P* < 0.01); †††Significant difference (*P* < 0.001) between HOX values at 100% NOX_max_ and 100% HOX_max_.

A two-way ANOVA test revealed significant oxygen concentration (*P* = 0.017, partial *η*^2^ = 0.243), time (*P* < 0.001, partial *η*^2^ = 0.917), and interaction effects (*P* = 0.043, partial *η*^2^ = 0.140) in the RMS amplitude of the EMG in the biceps brachii (Fig 2A). Multiple comparison testing showed that there was no significant difference between HOX and NOX values at 0% NOX_max_ (*P* = 0.058, *d* = 0.437) and 50% NOX_max_ (*P* = 0.026, *d* = 0.508); however, the HOX value was significantly lower than the NOX value at 100% NOX_max_ (*P* = 0.015, *d* = 0.569). Additionally, a paired *t*-test showed that the HOX value at 100% NOX_max_ was significantly lower than the HOX value at 100% HOX_max_ (*P* = 0.022, *d* = 0.538).

With respect to NIRS parameters (Fig 2B–2D), there was no significant oxygen concentration effect in any parameters (ΔO_2_Hb, *P* = 0.263, partial *η*^2^ = 0.059; ΔHHb, *P* = 0.611, partial *η*^2^ = 0.031; ΔcHb, *P* = 0.241, partial *η*^2^ = 0.065). A significant time effect was found in all parameters (ΔO_2_Hb, *P* < 0.001, partial *η*^2^ = 0.755; ΔHHb, *P* < 0.001, partial *η*^2^ = 0.622; ΔcHb, *P* < 0.001, partial *η*^2^ = 0.796). There was a significant interaction effect in ΔO_2_Hb (*P* < 0.001, partial *η*^2^ = 0.393) and ΔcHb (*P* < 0.001, partial *η*^2^ = 0.317), but not in ΔHHb (*P* = 0.269, partial *η*^2^ = 0.061). Multiple comparison testing showed no significant difference between HOX and NOX values at any measurement points for ΔcHb (0% NOX_max_, *P* = 0.242, *d* = 0.257; 50% NOX_max_, *P* = 0.209, *d* = 0.277; 100% NOX_max_, *P* = 0.031, *d* = 0.846), but the HOX value was significantly lower than the NOX value at 100% NOX_max_ in ΔO_2_Hb (0% NOX_max_, *P* = 0.271, *d* = 0.242; 50% NOX_max_, *P* = 0.882, *d* = 0.032; 100% NOX_max_, *P* = 0.003, *d* = 0.706). The HOX value at 100% NOX_max_ was significantly lower than the HOX value at 100% HOX_max_ in both ΔO_2_Hb and ΔcHb (ΔO_2_Hb, *P* < 0.001, *d* = 1.122; ΔcHb, *P* < 0.001, *d* = 1.070). There was no difference between the HOX values at 100% NOX_max_ and 100% HOX_max_ in ΔHHb (*P* = 0.246, *d* = 0.270).

There were statistically significant time (*P* < 0.001, partial *η*^2^ = 0.809) and interaction effects (*P* = 0.027, partial *η*^2^ = 0.165), but no significant oxygen concentration effect (*P* = 0.224, partial *η*^2^ = 0.073) in HR (Fig 2E). Multiple comparison testing showed no significant difference between HOX and NOX values at any measurement points (0% NOX_max_, *P* = 0.784, *d* < 0.001; 50% NOX_max_, *P* = 0.629, *d* = 0.367; 100% NOX_max_, *P* = 0.018, *d* = 0.469). There was a significant difference between HR under HOX at 100% NOX_max_ and that at 100% HOX_max_ (*P* = 0.003, *d* = 0.699). Blood lactate concentrations before starting the single-joint weight-lifting task were 1.1 ± 0.2 mmol/L under HOX and 1.1 ± 0.2 mmol/L under NOX; they were 2.5 ± 0.6 mmol/L and 2.3 ± 0.5 mmol/L after the task, respectively. A two-way ANOVA showed a significant time effect (*P* < 0.001, partial *η*^2^ = 0.935), but no oxygen concentration (*P* = 0.103, partial *η*^2^ = 0.107) or interaction effects (*P* = 0.327, partial *η*^2^ = 0.040).

**Fig 3:**
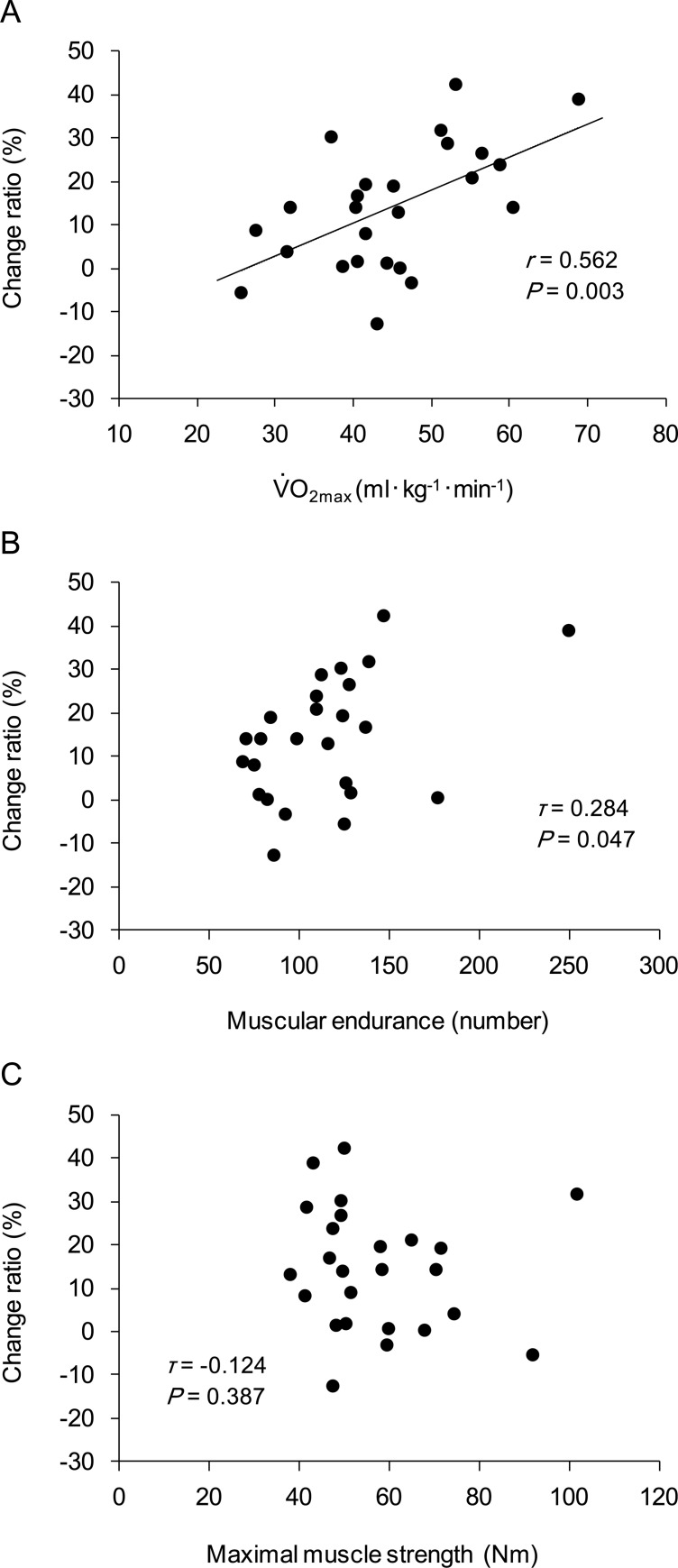
Physical fitness indices and percent change in maximum number of repetitions (n = 25). A, Relationship between maximal oxygen uptake (${\dot{V}O}_{2max}$) and percent change in maximum number of repetitions between hyperoxic conditions and normoxic conditions (change ratio). The change ratio was calculated as follows: (HOX_max_ − NOX_max_) / NOX_max_ × 100, where HOX_max_ and NOX_max_ indicate the maximum number of repetitions under hyperoxic and normoxic conditions, respectively. B, Relationship between muscular endurance and percent change in maximum number of repetitions. Maximum number of repetitions measured under normoxic conditions was used as an index of muscular endurance. The 95% confidence intervals (CI) for the correlation coefficient included 0 (95% CI = −0.003–0.565), indicating the absence of a significant correlation. C, Relationship between maximal muscle strength and percent change in maximum number of repetitions.

The percent change in the maximum number of repetitions between HOX and NOX was significantly correlated with the ${\dot{V}O}_{2max}$ (*r* = 0.562, 95% confidence intervals (CI) = 0.213–0.783, *P* = 0.003; Fig 3A). The 95% CI for the correlation coefficient between the percent change in the maximum number of repetitions and the maximum number of repetitions under NOX (muscular endurance) included 0 (*τ* = 0.284, 95% CI = −0.003–0.565, *P* = 0.047; Fig 3B); this indicated the absence of a significant correlation between these indices. The maximal isometric elbow flexion strength showed no significant correlation with the percent change in the maximum number of repetitions (*τ* = −0.124, 95% CI = −0.424–0.170, *P* = 0.387; Fig 3C).

## Discussion

This study aimed to investigate the effects of hyperoxia on muscular endurance during low-intensity single-joint repetitive muscle contractions until exhaustion, and to identify any individual differences in the effects in terms of physical fitness. The results indicated that hyperoxic conditions acutely improved low-intensity dynamic muscular endurance. Previous studies that showed the positive effects of hyperoxia on muscular endurance were conducted using maximal voluntary knee extensions [15,16] under higher inspired O_2_ fraction conditions (FiO_2_ = 1.00 at 1.3 ATA and FiO_2_ = 0.99 at 1.0 ATA) than those used in this study. Therefore, this study demonstrated that “low-intensity” single-joint repetitive muscle contractions could improve under “milder” hyperoxic conditions. This study further revealed individual differences in the effects. The degree of improvement had significant positive correlations with ${\dot{V}O}_{2max}$, indicating that individuals with higher whole-body endurance capacity achieved more benefits from hyperoxic conditions during dynamic muscular endurance exercise.

Our results demonstrated that the muscle activation level and ΔO_2_Hb were lower during the late stage of exercise under hyperoxic conditions. It is well known that the muscle activation level increases during submaximal muscle contractions until exhaustion [31,32]. The increase in muscle activation level with fatigue was confirmed under both the hyperoxic and normoxic conditions in this study. Furthermore, we demonstrated that the muscle activation level increased more gradually under hyperoxic conditions, suggesting that the development of fatigue was delayed under hyperoxic conditions. This finding agrees with that of Amann et al. [12], who reported that the integrated EMG of the vastus lateralis during submaximal bicycle pedaling was significantly lower under hyperoxic conditions (FiO_2_ = 1.00) than under normoxic conditions. Meanwhile, for the three subjects who achieved a smaller number of repetitions under hyperoxic conditions than normoxic conditions in this study, the RMS amplitude of the EMG increased with the number of repetitions in a similar manner under both conditions (when analyzed with a time course relative to HOX_max_ [i.e., at 0% HOX_max_, 50% HOX_max_, 100% HOX_max_], there was a significant time effect [*P* = 0.010, partial *η*^2^ = 0.898], but no oxygen concentration [*P* = 0.261, partial *η*^2^ = 0.547] or interaction [*P* = 0.360, partial *η*^2^ = 0.400] effects). Considering these results in those who either benefited or did not benefit from hyperoxia, delayed development of fatigue identified by EMG activity can be a significant physiological response in acute improvement of muscular endurance under hyperoxic conditions.

ΔO_2_Hb indicates changes in oxygenated hemoglobin and myoglobin concentrations and can be considered a reflection of the oxygen supply [33,34]. Therefore, a lower ΔO_2_Hb during the late stage of exercise under hyperoxic conditions may be interpreted as lower supply of oxygen. However, it must be noted that hyperoxic conditions can increase arterial dissolved oxygen due to the increased partial pressure of oxygen in inspired gas (Henry’s law) and that the ΔO_2_Hb only estimates the amount of oxygen binding to hemoglobin and myoglobin, but not arterial dissolved oxygen. Our preparatory experiment (see S1 Appendix) showed that the partial pressure of arterial oxygen (PaO_2_) significantly increased after 5-min inhalation of a gas mixture with an FiO_2_ of 0.30 during seated rest. Several previous studies also reported that hyperoxia increased CaO_2_ and PaO_2_ during exercise due to increases in arterial dissolved oxygen [4,12,13,18]. Considering these results, a 30-min pre-exposure and average 4.5-min task in an environment with an FiO_2_ of 0.30, as in this study, could increase the amount of arterial dissolved oxygen. Therefore, the lower ΔO_2_Hb found during the late stage of exercise under hyperoxic conditions does not necessarily indicate a lower supply of oxygen. Furthermore, from the beginning to the middle stage of exercise, ΔO_2_Hb and ΔcHb were not significantly different between hyperoxic and normoxic conditions. This can indicate an oxygen-rich environment in the muscles due to increased arterial dissolved oxygen under hyperoxic conditions, which might cause the results of the present study: increase in maximum number of repetitions and delayed elevation of muscle activation level due to fatigue.

The percent change in the maximum number of repetitions between hyperoxic conditions and normoxic conditions showed a significant positive correlation with ${\dot{V}O}_{2max}$ but no correlation with muscle strength. With regard to the correlation with muscular endurance, *P*-value was less than 0.05 while 95% CI of the correlation coefficient included 0 (the lower limit was −0.003). We recalculated the correlation coefficient after excluding two subjects with high muscular endurance values (Subject A, maximum number of repetitions 250; Subject B, maximum number of repetitions 177), because the correlation seemed to be affected by these subjects. The result showed a significant correlation (normally distributed, *r* = 0.483, 95% CI = 0.089–0.747, *P* = 0.019). Therefore, at this stage, we cannot determine whether individual muscular endurance is actually uncorrelated with the extent of the acute effects of hyperoxia on muscular endurance exercise. To summarize these results, the effect of hyperoxia on muscular endurance depends on the individual’s whole-body endurance capacity. Limiting factors in ${\dot{V}O}_{2max}$ are classified as both central and peripheral factors [23]. The central factors include pulmonary diffusion capacity, maximal cardiac output, and oxygen carrying capacity of the blood, whereas the peripheral factors include skeletal muscle characteristics. These central and peripheral factors contribute to the degree of muscular endurance [35]. It is conceivable that the peripheral rather than central factors caused the individual differences in the effects of hyperoxia on muscular endurance, since the HR after exhaustion was approximately 100 on average (it did not reach a near maximum) and did not differ between hyperoxic and normoxic conditions. The major components of the peripheral factors are capillary density and mitochondrial enzymes [23]. High capillary density confers an advantage in oxygen delivery to exercising muscles [36]. Increased mitochondrial enzymes exert metabolic effects that allow muscles to oxidize fat at a higher rate, which spares muscle glycogen and blood glucose during endurance exercise, and decrease lactate production during exercise [23]. During exercise under hyperoxic conditions, individuals with greater whole-body endurance capacity can extract more oxygen from the blood due to the high capillary density. This oxygen-rich condition in exercising muscles, in combination with increased mitochondrial enzymes, can generate metabolic benefits that are considered to be possible underlying mechanisms of the effects of hyperoxia, i.e., attenuated degradation of phosphocreatine [14], reduced muscle glycogenolysis [10], and faster phosphocreatine resynthesis [15].

This study had limitations. First, we excluded three participants who performed a greater number of repetitions until exhaustion under normoxic conditions from the comparisons of physiological indices during the repeated weight-lifting task between hyperoxic and normoxic conditions. After excluding these three participants, we could only estimate the physiological responses when participants were improving their muscular endurance acutely under hyperoxic conditions, but we could not estimate the physiological responses of participants who negatively responded to hyperoxic conditions. To elucidate the mechanisms underlying the effects of hyperoxia on muscular endurance exercise, it is necessary to perform a detailed examination of physiological responses in a large number of negative responders. Second, this study lacked a direct estimation of fatigue based on force output. To examine the effect of hyperoxia on muscular endurance exercise more precisely, the extent of fatigue based on force output should be measured along with physiological indices.

A recent study demonstrated that muscular endurance training, rather than intensive strength training, decreased the number of senescence-prone T cells in elderly individuals [37], indicating a potential effect of muscular endurance training on immune function. Muscular endurance is necessary for not only sports performance but also activities of daily life, particularly in the elderly; therefore, muscular endurance training may become more important as the world’s population ages. The results of the present study show that hyperoxic conditions can be applied to increase the number of repetitions until exhaustion during muscular endurance training. Individuals with higher endurance capacity can achieve more benefits, i.e., they can increase the number of repetitions when performing this type of training. This study further showed that even mild hyperoxic conditions (FiO_2_ = 0.30) could improve muscular endurance acutely. Such conditions are feasible in the context of a practical training environment. The system for creating hyperoxic conditions is similar to that for creating hypoxic conditions (with an oxygen generator substituting a nitrogen generator). If future studies further demonstrate the efficacy of exercise training under hyperoxic conditions, systems to create hyperoxic conditions will become more accessible, similar to the normobaric hypoxic chambers currently in widespread use in experimental and exercise training facilities.

## Conclusions

This study demonstrated that the maximum number of repetitions in one-arm dumbbell preacher curls at an intensity of 30% 1-RM acutely increased under hyperoxic conditions. The results suggested that hyperoxic conditions acutely improved low-intensity dynamic muscular endurance. The improvement was accompanied by delayed increase in muscle activation due to fatigue. Furthermore, the effects of hyperoxia on muscular endurance were positively correlated with whole-body endurance capacity.

## Supporting information

S1 Appendix Increase in partial pressure of arterial oxygen by five-minute inhalation of 30% oxygen. (DOCX)
