# Supplementary material for: Effects of hyperoxia on dynamic muscular endurance are associated with individual whole-body endurance capacity
Source: PLoS One. 2020 Apr 21;15(4):e0231643. doi: 10.1371/journal.pone.0231643 (PMC7173853; doi:10.1371/journal.pone.0231643)
Supplement: S1 Appendix — (DOCX) [file pone.0231643.s001.docx]

**S1 Appendix. Increase in partial pressure of arterial oxygen by five-minute inhalation of 30% oxygen.**

**Purpose**

This preparatory experiment aimed to investigate effect of 5-min inhalation of 30% oxygen (O_2_) on partial pressure of arterial oxygen (PaO_2_).

**Materials and methods**

Fourteen young men participated in this study. The age, height, and mass of the participants were 24 ± 1 years, 173.4 ± 6.7 cm, and 63.4 ± 8.3 kg (means ± standard deviations [SD]), respectively. This study was approved by the Research Ethics Committee of the University of Tokyo (Approval No. 12-28) and written informed consent was obtained from all participants. While sitting on a chair, participants breathed a hyperoxic gas mixture (30% O_2_ and 70% nitrogen) from a Douglas bag for 5 min. Approximately 100 μL of blood was taken from left and right fingertip before and after 5-min inhalation, respectively. PaO_2_ of the blood sample was measured using a blood gas analyzer (ABL80 FLEX CO-OX; Radiometer, Copenhagen, Denmark). A paired *t*-test was used to test difference in PaO_2_ between blood samples taken before and after 5-min hyperoxic gas inhalation. *P* < 0.05 was considered statistically significant.

**Results**

PaO_2_ after 5-min inhalation (112.4 ± 10.0 mmHg) was significantly larger than that before inhalation (82.3 ± 4.2 mmHg; *P* < 0.001).

**Conclusion**

PaO_2_ increases through 5-min inhalation of 30% O_2._
